# The Effects of Alternate-Day Corticosteroids in Autoimmune Disease Patients

**DOI:** 10.1155/2020/8719284

**Published:** 2020-05-18

**Authors:** Genny Margarita Chaia-Semerena, María Eugenia Vargas-Camaño, Cesar Daniel Alonso-Bello, Jorge Javier Guillén-Toledo, Ricardo Leopoldo Guido-Bayardo, Fernando Lozano-Patiño, Mariano Daniel Temix-Delfín, María Isabel Castrejón-Vázquez

**Affiliations:** Clinical Immunology and Allergy Service, National Medical Center 20 de Noviembre, Instituto de Seguridad y Servicios Sociales de Los Trabajadores del Estado, México City, Mexico

## Abstract

**Introduction:**

Several studiesdemonstrated that the use of alternate-day corticosteroid therapy maintains control of autoimmune diseases due to the prolongation of their therapeutic effect beyond their metabolic effect, with a significant decrease in side effects in patients. For this reason, the current recommendation for the use of these medications is in a short cycle to avoid adverse effects when used frequently and for prolonged periods of time.

**Objectives:**

To learn variations in serum levels of autoantibodies in autoimmune diseases treated with steroids on alternate days, as well as whether there are differences in the response to them depending on the type of disease. *Study Design*. A descriptive, retrospective, and cross-sectional study was conducted in which serum autoantibody levels were compared at the time of diagnosis and three months after alternate-day corticosteroid therapy.

**Results:**

We included 106 patients from three autoimmune connective tissue diseases (systemic lupus erythematosus, Sjögren syndrome, and Hashimoto's thyroiditis) and observed a statistically significant decrease in serum autoantibody levels both in patients with lupus and those with Hashimoto's thyroiditis, regardless of the sex of the patients, as well as the type of steroids used.

**Conclusions:**

Treatment with alternate-day corticosteroids achieved a statistically significant decrease in serum autoantibody levels in patients with systemic lupus erythematosus and Hashimoto's thyroiditis.

## 1. Introduction

Autoimmune diseases are characterized by the loss of control in a T lymphocyte subgroup with alteration in the differentiation of the own and the foreign [1–4]. When a secondary activation of lymphocytes is associated with the production of antibodies against various antigens of the body, it is called humoral autoimmune disease and, in this case, the treatment involves providing drugs that stop the uncontrolled production of autoantibodies to prevent the perpetuation of organ damage. The medications used for this purpose could trigger side effects that deteriorate the quality of life of patients and are usually dose dependent. Within these, the most used are systemic steroids [5–11]. Several publications have been demonstrated that the use of alternate-day corticosteroid therapy maintains control of autoimmune diseases due to the prolongation of their therapeutic effect beyond their metabolic effect, with a significant decrease in side effects [12–14]. It has been proven that steroid schemes on alternate days have a lower suppressive effect of the hypothalamic-pituitary-adrenal axis compared to treatment with daily intake when evaluated by means of insulin-induced hypoglycemia [15]. There is also known that the steroid scheme on alternate days administered to children with nephrotic syndrome does not affect the expected growth for age [16]. The concentration of the medication on alternate days maintains the serum levels and is associated with reduced suppression of the hypothalamic-pituitary-adrenal axis [17]. The current recommendation for the use of systemic steroids is in short cycles and accompanied by other steroid-saving drugs to reduce the side effects of prolonged cycles. In this article, we presented the response observed in serum levels of autoantibodies in patients with autoimmune diseases treated with alternate-day corticosteroid therapy between January 2008 and January 2013 at the Clinical Immunology and Allergy Service of the National Medical Center 20 de Noviembre, ISSSTE, Mexico.

## 2. Materials and Methods

A descriptive, retrospective, and cross-sectional study was conducted in which serum autoantibody levels were compared at the time of diagnosis and three months after alternate-day corticosteroid therapy. In the period between January 1, 2008, and January 1, 2013, 318 patients were evaluated for three autoimmune diseases encoded in the ICD-10, and the diseases were systemic lupus erythematosus (SLE), Sjögren's syndrome (SS), and Hashimoto's thyroiditis (HT). 106 patients were selected by the inclusion criteria, selecting those who had at least two autoantibody determinations, one of them prior to steroid treatment on alternate days after the diagnosis of autoimmune disease and the other three months after onset and without another immunosuppressive treatment that could interfere with the immune response. The dose of the steroid used was 0.5–1 mg/kg, calculated based on prednisone or its equivalent dose with deflazacort, and the decision to start treatment was the clinical activity of the autoimmune disease. Groups according to the autoimmune disease were classified. The autoantibodies selected for the diseases studied were anti-ds DNA (double-stranded DNA autoantibodies) for SLE, anti-SSA and anti-SSB for SS, and anti-Tg and anti-TPO (thyroglobulin and thyroid peroxidase) for HT. Subsequently, within the groups formed, they were subclassified according to gender, age, steroid indicated (prednisone or deflazacort), and dose. All autoantibody quantifications were performed in the immunology laboratory by the radioimmunoassay. The statistical analysis was performed in STATISTICA 8.0 and STATA 11 with a descriptive and inferential statistical analysis, and comparisons were made regarding the variables studied (disease, sex, and age) and steroids used. To investigate the strength of association between the nominal variables with a 95% confidence interval, we used an ANOVA analysis of one, two, and three ways, to compare the means of serum antibody levels before and after initiation of systemic steroid treatment. This retrospective study was approved by the CMN20NOV Institutional Review Board and Ethics Committee.

## 3. Results

A total of 106 patients were analyzed, 17 with SLE, 10 with SS, and 79 with HT with a mean age of 42.5 ± 17.04 years. The main features are summarized in Table 1.

Regarding the treatment used due to illness, the distribution was as follows: in SLE, 58.82% (*n* = 10) with prednisone and 41.18% (*n* = 7) with deflazacort. In SS, 50% (*n* = 5) with prednisone and 50% (*n* = 5) with deflazacort. Finally, in the HT group, 46.84% (*n* = 37) with prednisone and 53.16% (*n* = 42) with deflazacort.

**Figure 1:**
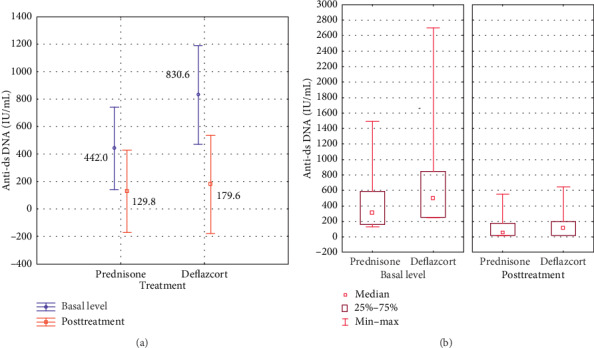
(a) Variance analysis of the mean in anti-ds DNA antibodies before and after steroid treatment. (b) Comparative analysis of the median in response of serum autoantibody levels, ranges, and interquartile ranges according to the indicated treatment and titration phase. Follow-up was done three months after the initial treatment.

The analysis of results in patients with SLE shows an average of anti-ds DNA antibodies prior to treatment with prednisone of 442.0 IU/mL and deflazacort 830.6 IU/mL and posttreatment of 129.8 IU/mL with prednisone and 179.6 IU/mL with deflazacort. The analysis of variance shows *p*=0.1851 for the difference in antibody levels between treatments and *p*=0.005 for the difference in pre- and posttreatment values independent of the indicated treatment (Figure 1).

**Figure 2:**
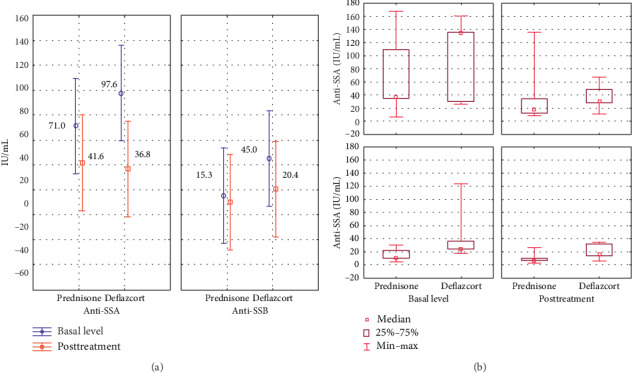
(a) Variance analysis of the mean in anti-SSA and anti-SSB antibodies before and after steroid treatment. (b) Comparative analysis of the median response of the serum level of autoantibodies, ranges, and interquartile ranges according to the indicated treatment and titration phase. Follow-up was done three months after the initial treatment.

For SS, two antibodies, anti-SSA and anti-SSB, were analyzed. The mean pretreatment values were as follows: for anti-SSA, 71.0 IU/mL with prednisone and 97.6 IU/mL with deflazacort, and for anti-SSB, 15.3 IU/mL with prednisone and 45.0 IU/mL with deflazacort. After treatment, the values for anti-SSA were 41.6 IU/mL with prednisone and 36.8 IU/mL with deflazacort. For anti-SSB, the values were 10.0 IU/mL for prednisone and 20.4 IU/ml for deflazacort. The analysis of variance shows *p*=0.2546 for the difference in antibody levels between treatments and *p*=0.0311 for the difference of pre- and posttreatment values independent of the indicated treatment. There is a greater difference between the decrease in anti-SSA antibodies compared to anti-SSB levels with *p*=0.0061 (Figure 2).

**Figure 3:**
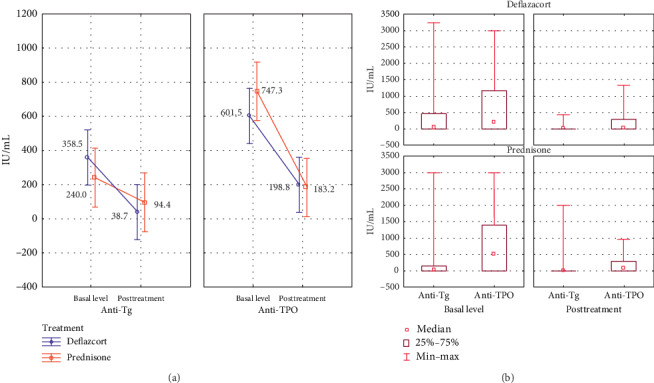
(a) Variance analysis of the mean in anti-Tg and anti-TPO antibodies before and after steroid treatment. (b) Comparative analysis of the median response of the serum level of autoantibodies, ranges, and interquartile ranges according to the indicated treatment and titration phase. Follow-up was done three months after the initial treatment.

For HT, two antibodies, anti-Tg and anti-TPO, were analyzed. The mean pretreatment value of anti-Tg was 240.0 IU/mL for prednisone and 358.5 UI/mL for deflazacort and of anti-TPO was 747.3 IU/mL for prednisone and 601.5 IU/mL for deflazacort. Posttreatment values are as follows: for anti-Tg, the value was 94.4 IU/mL for prednisone and 38.7 IU/mL for deflazacort and for anti-TPO, the value was 183.2 IU/mL for prednisone and 198.8 IU/mL for deflazacort. The analysis of variance shows *p*=0.7783 for the difference in antibody levels between treatments and *p* ≤ 0.0001 for the level of pre- and posttreatment antibodies. There is an important difference in the decrease between the types of antibodies, and the decrease among the anti-TPOs was greater compared to the anti-Tg with *p*=0.000038 (Figure 3).

## 4. Discussion

In the group of patients studied, three characteristic autoimmune pathologies (SLE, SS, and HT) were included; the number of patients was higher in the HT group due to the greater frequency of attention of this pathology in our service. It was evidenced in the results that there is a decrease in serum levels of autoantibodies in the three diseases regardless of the type of steroids used since the comparison between prednisone and deflazacort showed no statistical significance. The decrease of autoantibodies is seen in the study in the posttreatment of the diseases compared to the pretreatment.

There is sufficient evidence that demonstrates the safety in the use of alternate-day corticosteroid therapy in patients who may need long-term treatment with these medications. Suda et al. demonstrated that there is a 50% decrease in the infection rate in patients with rheumatoid arthritis with intake of steroids in alternate day versus the patients with daily intake for a period of one year [18]. There is no published evidence that objectively checks whether the steroid scheme on alternate days directly modifies the serum level of autoantibodies in patients with autoimmune diseases, so our results show that treatment on alternate days serves its purpose.

This clinical practice is based on basic immunological principles described since 1960, in articles published by several researchers and which unfortunately seem to be forgotten despite the results they shown.

In spite of the favourable results observed in this study, we consider it very important to complement with other studies that assess the clinical evolution of patients since it has been shown in other publications that with alternate-day corticosteroid therapy, the patients present less adverse effects.

The main reason for proposing the administration of steroids on alternate days is to reduce the prevalence of side effects including complications associated with suppression of the pituitary-hypothalamus-adrenal axis, improving the quality of life of patients with autoimmune diseases.

It is important to mention that the clinical activity of autoimmune disease does not correlate directly with serum levels of autoantibodies although these are the objective expression of autoimmunity [19].

## 5. Conclusion

Serum levels of autoantibodies in SLE, SS, and HT decrease significantly with alternate-day corticosteroid therapy. It is necessary to carry out comparative studies between daily and alternate day schemes jointly assessing the adverse effects, the decrease of autoantibodies, and the clinical activity of autoimmune disease. The use of steroids on alternate days instead of daily administration demonstrates a decrease in the adverse effects of the treatment and also a decrease in serum levels of autoantibodies.

## Figures and Tables

**Table 1:** General characteristics of the patients and classification by diagnosis.

Disease	*N*	Women	Men	Age range	Middle age
*Demographic characteristics*					
SLE	17	16	1	11–69	42.5 ± 17.04
Sjögren syndrome	10	9	1	60–94	69.8 ± 10.11
Autoimmune thyroid disease	79	67	12	11–72	46.3 ± 15.9

## Data Availability

The research ethics committee restricts the patient's data used to support the findings of this study in order to protect patient privacy according to The Federal Law on Protection of Personal Data in Mexico. Data are available from Maria Eugenia Vargas Camaño (dra.maruvargascam@gmail.com) for researchers who met the criteria for access to confidential data.

## Abbreviations

Anti-ds DNA: Double-stranded DNA autoantibodies
Anti-Tg: Anti-thyroglobulin autoantibodies
Anti-TPO: Anti-thyroid peroxidase autoantibodies
SLE: Systemic lupus erythematosus
SS: Sjögren syndrome
HT: Hashimoto's thyroiditis.

## Additional Points

(1) What is already known about this topic? The alternate-day corticosteroid therapy reduces the adverse effects in the long-term treatment compared to the daily dose schedule. (2) What does this article add to our knowledge? The evidence of decreased serum autoantibody levels with treatment for three months. (3)How does this study impact current management guidelines? The relevance of this paper lies in being able to recommend this treatment as an alternative to short steroid cycles in autoimmune diseases that require it.

## References

[1] Cohn M. (2009). On the opposing views of the self-nonself discrimination by the immune system. *Immunology & Cell Biology*.

[2] Sawla P., Hossain A., Hahn B. H., Singh R. P. (2012). Regulatory t cells in systemic lupus erythematosus (SLE); Role of peptide tolerance. *Autoimmunity Reviews*.

[3] Invernizzi P., Gershwin M. E. (2009). The genetics of human autoimmune disease. *Journal of Autoimmunity*.

[4] McHugh R. S., Shevach E. M. (2002). The role of suppressor t cells in regulation of immune responses. *Journal of Allergy and Clinical Immunology*.

[5] Miller A. V., Ranatunga S. K. M. (2012). Immunotherapies in rheumatologic disorders. *Medical Clinics of North America*.

[6] Grammatikos A. P., Tsokos G. C. (2012). Immunodeficiency and autoimmunity: lessons from systemic lupus erythematosus. *Trends in Molecular Medicine*.

[7] Yildirim-Toruner C., Diamond B. (2011). Current and novel therapeutics in the treatment of systemic lupus erythematosus. *Journal of Allergy and Clinical Immunology*.

[8] Spies C. M., Strehl C., Van Der Goes M. C., Bijlsma J. W. J., Buttgereit F. (2011). Glucocorticoids. *Best Practice & Research Clinical Rheumatology*.

[9] Sarnes E., Crofford L., Watson M., Dennis G., Kan H., Bass D. (2011). Incidence and US costs of corticosteroid-associated adverse events: a systematic literature review. *Clinical Therapeutics*.

[10] McDonough A. K., Curtis J. R., Saag K. G. (2008). The epidemiology of glucocorticoid-associated adverse events. *Current Opinion in Rheumatology*.

[11] Poetker D. M., Reh D. D. (2010). A comprehensive review of the adverse effects of systemic corticosteroids. *Otolaryngologic Clinics of North America*.

[12] Huscher D., Thiele K., Gromnica-Ihle E. (2009). Dose-related patterns of glucocorticoid-induced side effects. *Annals of the Rheumatic Diseases*.

[13] Hoes J. N., Jacobs J. W. G., Boers M. (2007). Eular evidence-based recommendations on the management of systemic glucocorticoid therapy in rheumatic diseases. *Annals of the Rheumatic Diseases*.

[14] MacGregor R. R., Sheagren J. N., Lipsett M. B., Wolff S. M. (1969). Alternate-day prednisone therapy. *New England Journal of Medicine*.

[15] Carter M. E., James V. H. (1972). Effect of alternate-day, single-dose, corticosteroid therapy on pituitary-adrenal function. *Annals of the Rheumatic Diseases*.

[16] Abbas M., Sham-Una U., Rambod T., Niloofar H., Ali R., Hadi Z. M. (2011). The effect of long-term steroid therapy on linear growth of nephrotic children. *Iranian Journal of Pediatrics*.

[17] Morris H. G., Neuman I., Ellis E. F. (1974). Plasma steroid concentrations during alternate-day treatment with prednisone. *Journal of Allergy and Clinical Immunology*.

[18] Suda M., Ohde S., Tsuda T., Kishimoto M., Okada M. (2018). Safety and efficacy of alternate-day corticosteroid treatment as adjunctive therapy for rheumatoid arthritis: a comparative study. *Clinical Rheumatology*.

[19] Garber K. (2014). Immunology: a tolerant approach. *Nature*.

